# Ultralow-Resistance High-Voltage Loaded Woven Air Filter for Fine Particle/Bacteria Removal

**DOI:** 10.3390/polym17131765

**Published:** 2025-06-26

**Authors:** Weisi Fan, Sanqiang Wei, Ziyun Zhang, Lulu Shi, Jun Wang, Wenlan Hao, Kun Zhang, Qiuran Jiang

**Affiliations:** 1 Key Laboratory of Textile Science & Technology, Ministry of Education, College of Textiles, Donghua University, Shanghai 201620, China; 2180053@mail.dhu.edu.cn (W.F.);; 2 Department of Technical Textiles, College of Textiles, Donghua University, Shanghai 201620, China; 3 Beijing Jinmao Living Environment Technology Co., Ltd., Beijing 102629, China

**Keywords:** ultraloose woven filter, high voltage loading, ultralow pressure drop, cleanable, indoor air filtration

## Abstract

Conventional filters for air filtration typically feature compact nonwoven structures, which not only lead to high pressure drop, significant energy consumption, and a decay in filtration efficacy, but are also uncleanable, resulting in substantial pollution upon disposal. In this study, filters with high-voltage electrostatic loading capability were developed with a dopamine binding layer to facilitate the establishment of an Ag conductive layer on the surface of ultraloose woven structure fabrics (pore size: 73.7 μm). The high-voltage-loaded woven structure filtration (VLWF) system was constructed with a negative-ion zone, a high-voltage filtration zone, and a grounded filter. The morphological, chemical, and electrical properties of the filters and the filtration performance of the VLWF system were evaluated. The single-pass filtration efficiencies for PM_2.5_ and *E. coli* were 67.4% and 97.0%, respectively. Notably, the pressure drop was reduced to 6.2 Pa, and the quality factor reached 0.1810 Pa^−1^ with no detectable ozone release. After three cycles of ultrasonic cleaning, approximately 58.4% of filtration efficiency was maintained without any increase in air resistance. The removal of PM_2.5_ and microorganisms by this system was not solely reliant on blocking and electrostatic attraction but may also involve induced repulsion and biostructure inactivation. By integrating the ultraloose woven structure with high-voltage assistance, this VLWF system effectively balanced the requirements for high filtration efficacy and low air resistance. More importantly, this VLWF system provided a cleanable filter model that reduced the pollution associated with conventional disposable filters and lowered costs for customers.

## 1. Introduction

Given that over 80% of modern life is spent indoors, indoor air pollutants, including nano-, submicro-, and micro-particulate matter (PM), and pathogenic microorganisms, pose serious threats to public health [1,2,3,4,5,6,7,8]. Thus, the effective removal of indoor air pollutants is in growing demand. Air filtration technologies have been widely employed for indoor air quality control [9]. The filtration performances highly depend on the properties of the filters [10]. Fibrous nonwoven filters account for more than 70% of the air filter market [2,11]. Conventional nonwoven filters provide the air purification function based on mechanical blocking (interception, inertia impaction, Brownian diffusion) [12], which elicits the intrinsic conflict between low air resistance, small energy consumption, long service life, and high filtration efficiencies [13]. To achieve effective filtration, dense or thick filter structures are required, but inevitably generate fast clogging and high pressure drop, which cost up to 50% of the energy consumption of the driving fan [14]. Since the elevation of massive energy efficiency is gaining increasing attention to solve the climate emergency, significant efforts have been devoted to developing more effective and energy-saving filtration systems.

The incorporation of electrostatic assistance into filtration systems is considered to be the most effective approach to retain or elevate filtration efficiency and reduce air resistance [2,15,16]. The electrostatic assistance can be introduced passively or actively. In a typical passive electrostatic filtration system, electret filters made from fibers with high dipole charging (corona charging [17], triboelectric charging [18], or induction charging) and provide additional electrostatic attraction to air pollutants without a continuous energy supplement [19,20,21,22]. With higher efficient filtration efficacy, the electret filters allow a looser or thinner filter structure compared to the conventional compact nonwoven filters and produce lower air resistance. However, electret-dust interactions are based on weak and unstable electric dipole-induced-dipole interactions, resulting in low dust-holding capacity, limited particle capture distance, reduced attraction force, along with dust accumulation and charge decay during a long service duration [23]. While triboelectric charging offers environmental advantages, particulate accumulation on friction layers induces interfacial blockage, compromising sustained operational stability [18].

The active-charged filtration systems have been proposed as alternatives to overcome the shortcomings of passive-charged filters. Most active-charged filtration systems consist of two regions: the particle charging region with discharge electrodes and the particle collecting region with grounded collection electrodes. During filtration, the particles are pre-charged in the first region and captured in the second region. Various types of active filtration systems have been reported, and they are mainly dependent on ionizing, such as electrostatic precipitators (ESPs) [24] and ionizer-assisted filtration systems [25]. In the particle charging region, tungsten wires are usually employed, but maintaining the delicate wires requires specific equipment and trained employees [26]. In the particle capturing region, if the collectors are parallel to the airway, the removal of fine particles is less efficient; if the collectors are vertical to the airway, high air resistance and frequent replacement of nonwoven collection filters are concerns [27]. A desirable filtration system is expected to be effective in filtration efficiency, low in air resistance and energy consumption, long in serving life, stable with power supply, and with a low-cost and convenient renewal process.

Primary methods for conductive filter modification encompass vacuum deposition techniques (such as sputtering and physical vapor deposition), electroplating, and electroless plating [28]. While vacuum deposition yields high uniformity, it necessitates expensive apparatus, rendering it viable only for small-batch, high-value products. Electroplating exhibits low deposition efficiency and requires a pre-sputtering step [29]. Electroless plating, conversely, offers distinct advantages in process simplicity, cost-effectiveness, and environmental compatibility, making it ideal for large-area substrates [30]. Polydopamine (PDA) facilitates uniform conductive layer deposition on textiles by exploiting its bioinspired adhesive properties and metal-chelating capability (via catechol/quinone groups) [31,32,33]. Owing to its operational simplicity, low cost, and eco-compatibility, PDA-mediated modification represents a highly advantageous approach for functional coatings on large-area fabrics. The integration of PDA with electroless plating enables the scalable production of conductive textiles, thereby establishing a foundation for industrial implementation [34].

Aimed to achieve the above requirements, this work proposed a highly efficient and low-resistance active-charged filtration system composed of a negative ionizer for pre-charging and a pair of conductive ultraloose gridding woven filters for dust capture. A silver (Ag) conductive layer was established on the filament surfaces of the polyethylene terephthalate (PET) woven grid base with the assistance of a PDA binding layer. The front gridding woven filter was supplied with positive high voltages (HV), and the back one was grounded. Two electrostatic fields were established among the three parts. Compared to conventional coarse or ultrafine-fiber-based nonwoven filters, the gridding woven filters in the active-charged filtration system were expected to provide comparable filtration performance, ultralow resistance and energy consumption, washability, and high mechanical properties. The filtration performances of PM_2.5_ and bacteria were investigated to confirm the potential of this filtration system for indoor air cleaning. Based on simulation and dust accumulation observation, the mechanisms for removing PM_2.5_ and bacteria were deduced.

## 2. Materials and Methods

### 2.1. Filter Preparation

**Figure 1:**
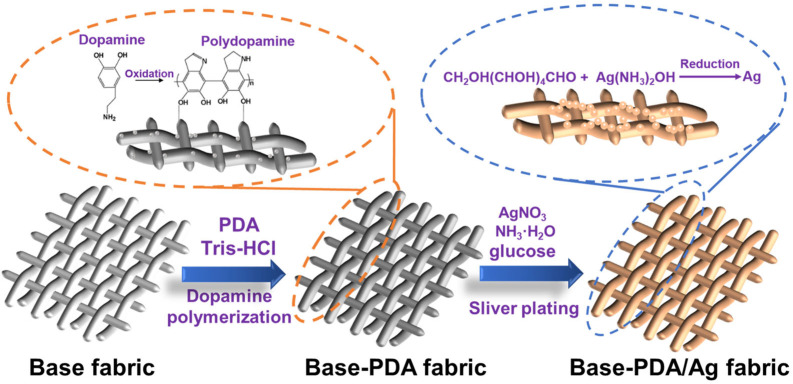
Modification process of filters.

The filter preparation process is described in Figure 1. Woven and nonwoven filters were prepared for comparison. The nonwoven and gridding woven PET fabrics were selected as the base substrates and noted as NWFL (55 g/m^2^), NWFH (113 g/m^2^), WFL (35 g/m^2^), and WFH (43 g/m^2^). The PDA binding layer was established on the filaments of fabrics by treating in the dopamine bath (DOPA, 2 g/L, pH 8.5) at 30 °C for the designed durations and labeled as “Fabric index-PDA”. The silver (Ag) conductive layer was further constructed on the PDA binding layer. The DOPA-treated fabrics were immersed in the silver ammonia bath (10 g/L, pH 11, with 0.25 wt.% polyvinylpyrrole) and modified for 30 min at room temperature. The glucose solution (20 g/L) was then introduced dropwise. The final volume ratio of the glucose solution to the silver ammonia solution was 2:1. After the reaction for another hour with stirring, the modified fabrics were washed, dried, and noted as “Fabric index-PDA/Ag”.

### 2.2. Filter Characterization and Simulation

The macro- and micro-morphologies of filters were observed under a three-dimensional rotating light microscope (QHS-1000, Questar (China) Co., LTD., Suzhou, China) and a scanning electron microscope (SEM, TM3000, Hitachi, Tokyo, Japan). The fiber diameter of filters was measured from images using the software Image J (National Institutes of Health, Fiji version) [35]. Pore size of filters was analyzed using PMI Capillary Flow Porometer (CFP-1100A, Porous Material Inc., New York, NY, USA) based on the bubble-point method. The thickness of the filters was determined by a fabric thickness gauge (LFY-205, Shandong Academy of Sciences, Qingdao, China) with the average value calculated from five measurements under a pressure of 1.01 KPa. Following DOPA treatment, the surface chemical structures of the untreated and treated fabrics were analyzed via Fourier transform infrared spectrometer (FTIR, Nicolet 6700, Thermo Fisher Scientific Inc., Waltham, MA, USA) equipped with a universal diamond crystal attenuated total reflectance (ATR) accessory, and the FTIR spectra were recorded over a wavenumber range of 400 to 4000 cm^−1^ at a resolution of 4 cm^−1^. The WFH treated by DOPA for 24 h and the silver ammonia system for 30 min was scanned under an X-ray diffractometer (XRD, D/max-2550PC, Rigaku Corporation, Tokyo, Japan) with Cu-Kα radiation, performed with a 2θ range from 5° to 90° at an acceleration voltage of 40 kV and a current of 200 mA. The bursting strength and elongation were evaluated on a universal testing machine (YG026MB-250, Fangyuan Instrument, Wenzhou, China) using round samples (6 cm diameter) mounted on the sample frame with the spherical puncture head set at an initial position of 450 mm, tested at a puncture rate of 300 mm min^−1^ with five specimens prepared for each sample. The air permeability of each filter (20 cm × 20 cm) was assessed by a digital fabric permeability tester (YG461E, Fangyuan Instrument, Wenzhou, China), with each test conducted at 200 Pa and ten different spots randomly selected for testing, with five specimens prepared for each sample. The surface-specific resistances were measured, and the detailed testing process was presented in the Supplementary Materials. The antibacterial functions of the uncharged filters were also investigated using the agar diffusion plate method and the shake flask method. As shown in Figure S2, the filtration system was assembled by a carbon fiber brush negative ionizer (−3.5 kV, Yufeng Electronics Co., Ltd., Xiamen, China), a positive HV-loaded woven filter, and a grounding woven filter. The PM_2.5_ and microbial filtration performances were tested using the standard dust (PTI ISO 12103-1, A1 Ultrafine Test Dust, Powder Technology, Inc., Arden Hills, MN, USA) and *Escherichia coli* (*E. coli*) as the model substrates on the customized filtration testing system (Figure S2). The filtration stability of the cleaned filtration system was evaluated after ultrasonic washing. The reusability performance of the WFH-PDA/Ag filter was evaluated using a 2 wt.% NaCl aerosol under 40 kV. The filter underwent ultrasonic washing with deionized water for 5 min to assess its reusability. The antibacterial performance of filters with different structures was assessed using the agar diffusion plate method and the shake flask method against *E. coli*. The ozone generation of this system was measured at an HV of 20 kV. The electric fields established on the filters were simulated using the software of Solidworks (version 2018) and COMSOL Multiphysics (version 5.5). The detailed processes for the property evaluations of the filtration system and the electric field simulation were described in the Supplementary Materials.

## 3. Results and Discussion

### 3.1. Effects of DOPA Modification Duration on the Surface Morphological, Chemical, and Electrical Properties

**Figure 2:**
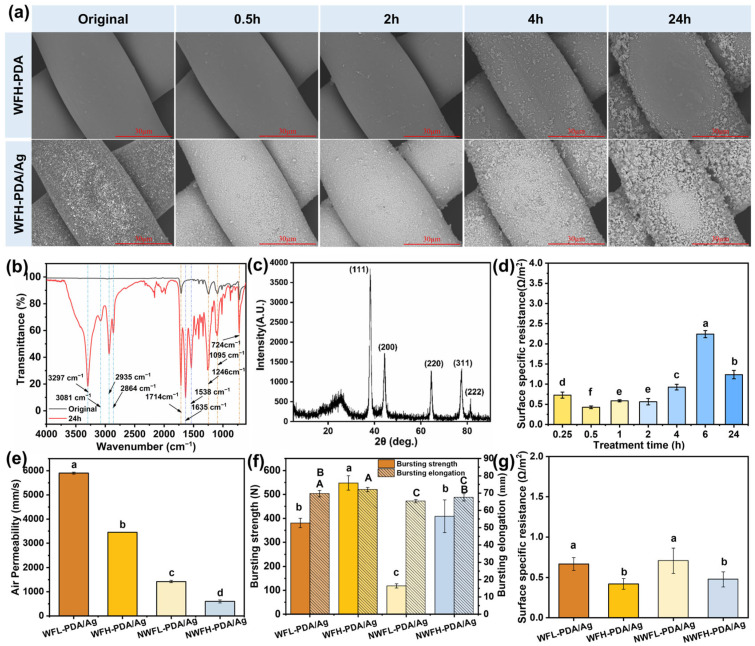
Effects of DOPA modification duration and base fabric structures on the basic properties of filters. (**a**) Surface morphologies of the WFH-PDA and WFH-PDA/Ag filters; (**b**) FTIR spectra of the WFH-PDA filters; (**c**) X-ray diffractogram of the WFH-PDA/Ag filter; (**d**) Surface specific resistances of the WFH-PDA/Ag filters; Air permeability (**e**), mechanical properties (**f**), and surface specific resistances (**g**) of filters with different base structures.

After DOPA modification, the PET fabrics changed from bright white to brown, and the color shades were shifted from light to dark as the treatment duration was extended (Figure S3a). The dark color was formed by the pseudo-melanin structure on polydopamine [36]. Treated for less than 2 h, the fibers exhibited smooth surfaces, but PDA clusters were agglomerated on filaments by prolonging the treatment. A longer treatment duration led to more deposited clusters with larger sizes (Figure 2a and Figure S3c). In the FTIR spectrum of the bare PET fabric (black curve, Figure 2b), the typical peaks of PET at 1715, 1240, 1094 and 720 cm^−1^ could be observed, which ascribed to the stretching vibration of -O-C=O-, -C-O and C-O-C, and the out-of-plane bending vibration of benzene ring [37,38]. After DOPA modification, the intensities of these PET peaks have been slightly weakened, and the peaks of PDA were observed. The peaks at 1635 and 1538 cm^−1^ stood for the stretching vibration of -C-C- and C=N in the indole ring [39,40]. The broad peak within the range of 3500–3200 cm^−1^ revealed the stretching vibration of O-H and N-H, and the distinct peaks at 3081 and 2935 cm^−1^ were attributed to the C-H stretching vibration of aromatic. These results indicated the successful establishment of the thin PDA binding layer on PET fibers.

After the silver nitrate treatment, the color of the filters turned from dark brown to light yellow (Figure S1b), and the Ag conductive layer was observed on the PDA binding layer (Figure S1d). Of note, limited Ag was anchored on the bare PET base, which proved that PDA coating provided effective assistance for Ag deposition. The negatively charged PDA layer could exert an attraction force on the positively charged silver ions and form a silver-ion-rich region to facilitate the reduction reaction (Figure 1). The reductive nature of PDA helped to maintain the elemental state of silver [41]. More Ag coating accumulated on the surface of the base fabrics treated by DOPA for longer. The X-ray diffractogram of the filter displayed the characteristic diffraction peaks located at 2θ = 38.3°, 44.3°, 64.6°, 77.6°, and 81.5° referring to the (111), (200), (220), (311) and (222) crystal planes of silver, and no peak representing oxide or halide of silver, which confirmed the successful coating of elemental Ag (Figure 2c) [42].

The three samples, WFH, WFH-PDA, and WFH-Ag, consistently exhibit high resistance. The WFH fabric base is non-conductive, and PDA is a non-conductive polymer binder. Consequently, the WFH-PDA sample maintained high resistance. Although silver is conductive, direct Ag deposition on WFH (WFH-Ag) yields discontinuous aggregates without a percolating network, maintaining high resistance. This morphological discontinuity (confirmed via SEM, Figure S3d—original) prevented effective electron transport, resulting in persistently high resistance for the WFH-Ag sample. These findings demonstrate that constructing the PDA binding layer is essential for depositing enough Ag to form a complete conductive layer with low resistance. The DOPA treatment for merely 15 min could provide the filter with a low conductivity of 0.73 Ω/m^2^ (Figure 2d). The lowest surface specific resistance of 0.43 Ω/m^2^ was achieved after treating for 0.5 h, indicating a full coverage of the Ag layer and the establishment of an effective electron transformation network (Figure 2a and Figure S1d). Prolonging the DOPA treatment duration increased the formation of PDA and Ag clusters, which led to a rise in surface-specific resistance. The maximum resistance was achieved at 2.24 Ω/m^2^ on the filter treated by DOPA for 6 h. This sample showed a rougher surface, and hence, longer electron conductive paths might be built with higher resistances. Interestingly, the filter modified for 24 h exhibited a reduced resistance of 1.24 Ω/m^2^. The possible reason might be that protruding PDA and Ag clusters above the filament surfaces established new conductive paths that shortened the lengths of conductive routes. In the following work, the DOPA treatment for 0.5 h was used.

### 3.2. Effects of Base Fabric Structure on the Permeability, Mechanical, and Electrical Properties of Filters

The base fabrics with four different structures were selected to reveal the influence of the fabric structure on filtration performance. The two woven filters have similar filament diameters, fabric thicknesses, and plain-woven structure, but different fabric densities (35 g/m^2^ for WFL and 43 g/m^2^ for WFH) (Figure S5). The WFL-PDA/Ag filter with large square pores (148.3 μm) displayed the highest air permeability of 5900.6 mm/s (Figure 2e), while the air permeability of the WFH-PDA/Ag filter with smaller pores (73.7 μm) decreased by 41.5% to 3451.3 mm/s. Compared to the woven filters, the nonwoven filters showed more packed structures with finer fibers (20 μm) and significantly higher thicknesses to achieve adequate mechanical properties (Figure S5b). Besides, small flat melts formed during the spun-bonding process could be observed to interlock the fibers (Figure S4). Thereby, the air permeabilities of the nonwoven filters were only 10.2% and 24.1% of the WFL-PDA/Ag filter’s value (Figure 2e). Since the permeability of filters could directly affect the pressure drop, the WFL-PDA/Ag filter with the highest permeability would be preferred.

Since filters are usually exposed to unidirectional blowing, the bursting strength plays a major role in the filters’ longevity. The bursting strengths of the WFH-PDA/Ag and WFL-PDA/Ag filters could achieve as high as 548.0 N and 380.6 N (Figure 2f). Noticeably, with about 69.0% lower fabric density, the WFL-PDA/Ag filter achieved similar bursting strength to the NWFH-PDA/Ag filter (408.7 N). This comparison indicated that the woven structure could create strong filters with fewer fibers and lower air resistance. Noticeably, the strength of a nonwoven filter highly depends on its density and thickness. A dramatic weakening in the bursting strength (71.0%) was observed when the fabric density of the nonwoven filter shifted from 113 g/m^2^ to 55 g/m^2^ and the fabric thickness reduced from 0.34 to 0.19 mm. The bursting elongations of the woven filters were slightly higher than those of the nonwoven ones, due to the inherent fiber slipping ability in the woven structure.

The filters possessed low surface specific resistances varying from 0.43 to 0.71 Ω/m^2^ (Figure 2g). Interestingly, the WFH-PDA/Ag filter with a low 43 g/m^2^ fabric density had the smallest surface specific resistance of 0.43 Ω/m^2^, similar to the resistance (0.48 Ω/m^2^) of the NWFH-PDA/Ag filter with the highest fabric density of 113 g/m^2^. Although the NWFH-PDA/Ag filter might provide more contact points among fibers in the whole filter, the contact points are not continuous, and the conductive paths are complicated due to the nonwoven structure. On the contrary, the woven fabrics were made up of regularly arranged warp and weft filaments, which aided in electron transfer through the shortest path in these two directions, resulting in low resistance with fewer fibers.

### 3.3. Effect of the Filter Structure on the Filtration Performance

**Figure 3:**
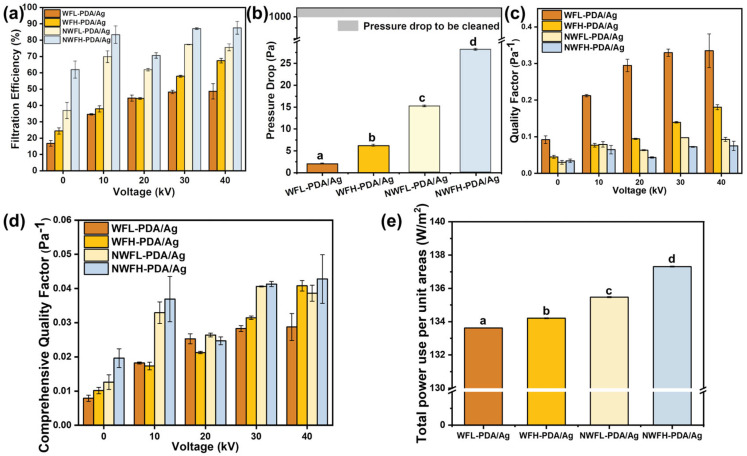
Effect of the fabric structure on the filtration performance. Filtration efficiency (**a**), pressure drop (**b**), quality factor (**c**), comprehensive quality factor (**d**), and total power use per unit area (**e**).

The filtration performance of the VLWF system is demonstrated in Figure 3. The filtration efficacies of the bare filters were primarily dependent on physical blocking, including the processes of diffusion, inertia, and interception [43]. The single-pass filtration efficiencies of the loose woven filters were merely 16.8 and 24.4%, and the efficiencies of compact nonwoven filters were 36.8 and 61.9%. By applying a positive high voltage of 10 kV, the filtration efficiencies of all filters could be promoted significantly. The filtration efficiencies of the nonwoven filters could achieve 69.8 and 83.3%, and the filters could be classified as high-medium grade [44]. The elevation ratios of nonwoven filters were 89.7 and 34.6%. The filtration efficiencies of the woven filters were improved to 34.6 and 37.9%, and the elevation ratios achieved 106.0 and 55.3%. Further enhancement in the applied voltage from 10 to 40 kV did not induce obvious elevation in the filtration efficiency of the nonwoven filters but improved the performance of the woven filters by 14.1 and 29.5%, reaching 48.7 and 67.4%, respectively, and elevated the filter grade from coarse to medium and high-medium grade, respectively.

**Figure 4:**
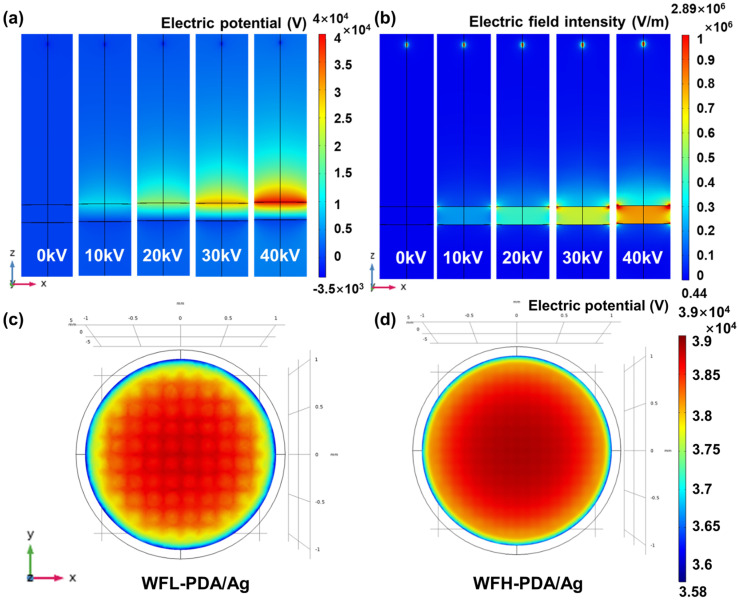
The side view of the simulations of electric potential and electric field intensity on the WFH-PDA/Ag filters supplied with positive charges ranging from 0 to 40 kV (**a**,**b**) and the top view of the simulations of electric field potentials on the WFL- and WFH-PDA/Ag filters at 40 kV (**c**,**d**).

**Figure 5:**
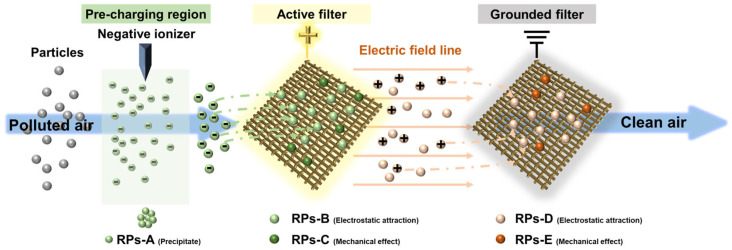
Filtration mechanism of the high-voltage aided system.

Figure 4a,b displays the simulations of electric potential and field intensity when positive high voltages (HVs) were applied to the active filter. The potential and intensity distributions can be divided into two regions. A weak electric field was established in front of the active filter, and the potential near the active filter was higher. A stronger and more uniform electric field was formed between the active and grounded filters. Both potential and intensity increased as the HV was elevated. The possible filtration mechanism is deduced and shown in Figure 5. Fine particles in the polluted air first pass through the region near the negatively charged ionizer. Some particles agglomerate and precipitate, and this part of the removed particles (RPs) is referred to as RPs-A (RPs-A). The remaining particles will likely acquire negative charges and are attracted to the positively charged active filter. In addition to mechanical blocking (RPs-C), the active filter captures particles through electrostatic adsorption (RPs-B). The escaped particles acquire positive charges and are attracted to the negatively charged grounded filters. They can be partially removed by attachment to the grounded filter or precipitation (RPs-D and E). Thus, the increase in HV facilitated the establishment of a stronger electrostatic field, exerting higher electrostatic forces to promote effective particle removal. Since the electric potential near the filaments is higher, the WFH-PDA/Ag filter with a denser structure can form a stronger electric field and exert a greater influence on particles. Therefore, the filtration efficiencies of the WFH-PDA/Ag filter at all HVs were slightly higher than those of the WFL-PDA/Ag filter.

Remarkably, it is important to highlight that as the intensity of the electric field around the positive filter increased, the halo near the edge of the filter transitioned from cyan green to a vivid red hue. This transformation suggested the occurrence of a phenomenon similar to that of an ionizer at the filter’s periphery. The particles passing through the front ionizer were negatively charged. Due to the potential difference, the positive filter exerted an attraction force on the negatively charged particles. As these particles approached the positive filter, some adhered to the surface of the filter, while others transitioned from a negative to a positive state by losing electrons. Once the remaining particles passed through the front positive filter, they entered the electric field between the filter and the grounded layer. The positively and negatively charged particles could agglomerate to form larger particles and precipitate, while the remaining particles might experience repulsive or attractive forces from the filter and the grid, depending on their charges. The elevation in the applied HV from 10 kV to 40 kV could enhance the intensities of electric fields from 3 cm to 10 cm (Figure 4a). Noticeably, at 20 kV, the filtration efficiencies of nonwoven filters were even reduced. Upon applying 20 kV, a slight decrease in negative ion concentration was observed, resulting in a decline in filtration efficiency. A further rise in HV might promote the generation of negatively charged particles, potentially enhancing filtration performance. This phenomenon was less pronounced when using loosely woven filters. Shifting the charges borne by the loosely woven filters may require significantly higher charges.

The pressure drop results exhibited an opposite trend compared to the air permeability data (Figure 3b). The thin woven filters with large pore sizes could achieve ultralow pressure drops of 2.0 Pa (WFL-PDA/Ag) and 6.2 Pa (WFH-PDA/Ag). However, when the filter structure shifted to the thick nonwoven filters with small pores, the pressure drops were elevated significantly to 15.2 Pa (NWFL-PDA/Ag) and 28.2 Pa (NWFH-PDA/Ag). The loaded HVs had minimal impact on the pressure drops of the filters.

To express the overall filtration performance, counting both filtration efficiency and air resistance, the quality factors (QF) of filters were calculated and shown in Figure 3c. The WFL-PDA/Ag filter exhibited superior filtration performance compared to other filters. The QF of the WFL-PDA/Ag filter was about 104.0% higher than the values of the rest of the filters without charging. Once the external voltages were supplied, the QF of the WFL-PDA/Ag filter was augmented by 130.7 to 264.0% and achieved 0.3350 Pa^−1^ at 40 kV. WFH-PDA/Ag filters can reach a QF of 0.1810 Pa^−1^ at 40 kV, which is 2.0~2.4 times that of NWFL and NWFH filters. Overall, the woven filters displayed obvious superiority, which benefited from their ultraloose structure and the assistance of the established electric field.

Along with the increasing attention to carbon emissions, the low energy consumption of a filtration system is emphasized. The comprehensive quality factors (CQF) of filters were calculated from their filtration efficiencies, pressure drops and power dissipations (details in Supplementary Materials) [14,45]. At a flow rate of 0.1 m/s, NWFH exhibits the highest CQF value at 0.0428 Pa^−1^ under 40 kV, while WFH follows closely with a value of 0.0408 Pa^−1^, representing 95.3% of the nonwoven CQF value (Figure 3d). This places it on par with the CQF of the high-density nonwoven filter. Conversely, NWFL occupies the third position in performance ranking, while WFL exhibits the least favorable performance. Through a significance analysis of the CQF values for WFH, NWFL, and NWFH, it was established that there were no statistically significant differences. This finding suggests that high-density WF filters are competitive with NWF filters when considering a comprehensive assessment of filtration efficiency, pressure drop, and power consumption. As a description of a product, the total power use per unit area (TPAs) of filters was also calculated (details in Supplementary Materials). By the sequence of fabric density, the TPA values exhibited an ascending trend: NWFH exhibited the highest value at 137.3 W/m^2^, followed by WFH at 134.2 W/m^2^, and the lowest value was observed in WFL at 133.6 W/m^2^, constituting a 97.3% nonwoven ratio (Figure 3e). The TPAs of commercial filters showing filtration efficiencies around 70 to 87% were approximately 180 to 300 W/m^2^ at flow rates around 0.05~0.13 m/s [14]. The comparison revealed that the application of woven filters could reduce the operation energy consumption by 46.4 to 166.4 W/m^2^. Besides, according to the standard of ASTM D6830-2002 [46], the pulse cleaning pressure of the pulse jet filters is 1000 Pa, which is almost 500 times the pressure drop of the WFL-PDA/Ag filter. It can be deduced that the single working cycle of these woven filters could be ultralong with extremely low cleaning frequency.

In addition to energy consumption, ozone emission is also a concern. Ozone production of the filtration system with the WFH-PDA/Ag filter was undetectable by the ozone tester, which indicated that the ozone concentration was far below 0.1 ppm, the upper limit required by the Occupational Safety and Health Administration [47].

### 3.4. Reusability of Voltage Loading Woven Air Filters

**Figure 6:**
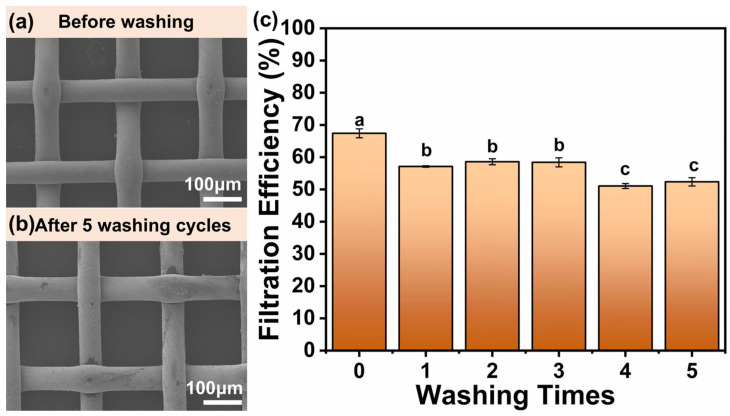
Reusability assessment of the WFH-PDA/Ag filter. The SEM images of the WFH-PDA/Ag filter’s morphology before and after the washing process (**a**,**b**). Filtration efficiency of WFH-PDA/Ag filter subjected to washing cycles ranging from 1 to 5 times (**c**).

After a single wash cycle, the filtration efficiency declined from 67.4% to 57.1%, indicating a reduction of 10.3%. As illustrated in Figure 6a, the PET layer was uniformly covered with the silver layer before washing. However, after washing, some portion of the silver layer detached from the surface, reducing the electrostatic action sites and consequently diminishing filtration efficiency. Notably, after 1 to 3 washing cycles, the filtration efficiency remained stable, fluctuating between 57.1% and 58.4%. This observation underscores the enduring reusability of the WFH-PDA/Ag filter.

### 3.5. Dust Accumulation Behaviors on Filters

**Figure 7:**
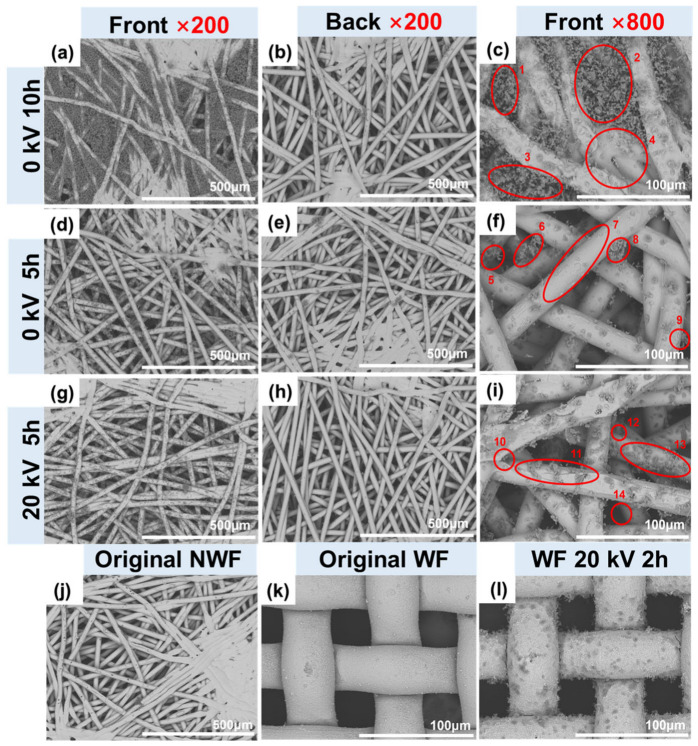
Dust accumulation on the NWFH- and WFH-PDA/Ag filters (**a**–**f**), the NWFH-PDA/Ag filter after the mechanical filtration for 5 or 10 h; (**g**–**i**) the NWFH-PDA/Ag filter after the filtration at 20 kV for 5 h; (**j**,**k**) the original NWFH- and WFH-PDA/Ag filters; (**l**) the WFH-PDA/Ag filter after the filtration at 20 kV for 2 h. The magnifications were set at 200× and 800×.

To facilitate the observation of dust accumulation behaviors, the NWFH- and WFH-PDA/Ag filters were selected for quick dust accumulation assessment. The operating conditions and testing procedures are detailed in the Supplementary Materials. After filtration, a greater amount of dust was trapped on the front sides of all the nonwoven filters compared to their backsides (Figure 7). In comparison to the uncharged filter, the charged filter exhibited similar or lesser particle accumulation on the front side (Figure 7d,g) and significantly fewer particles on the back side (Figure 7e,h).

In the absence of HV, dust tended to accumulate in the inter-fiber spaces, and fewer dust particles adhered to the fiber surfaces (Figure 7f). Dust clusters initially formed at the interlacing points between crossed fibers (Figure 7f Region 5, 6, 8, 9) and gradually filled the pores as the duration of filtration increased (Figure 7c Region 1–3). Upon the application of HV, dust clusters preferentially accumulated on fiber surfaces in a dendritic shape, regardless of whether the filter was a nonwoven or woven (Figure 7i,l). Enze Tian et al. observed a similar phenomenon in a dielectric hetero-caking filter within an electrostatic field [48]. In their study, particles were positively charged in a pre-charging zone and subsequently moved to the filtration zone with a dielectric filter in a parallel external electrostatic field. This filter induced negative charges on the upstream side. As recorded by the charged-coupled device (CCD) camera, these particles were deposited on fiber surfaces at an accelerated rate, forming particle chains that protruded perpendicularly from the fibers. These particle chains resemble the dust clusters observed on the charged filters. They proposed that the capture of positively charged particles was owing to the electric force between the particles and fibers. This mechanism could be one of the filtration processes in our system, but it may not be the only one. Interestingly, when calculated based on the filtration efficiencies at 20 kV, the captured dust on the uncharged NWFH-PDA/Ag filter for 5 h was 11.0 mg, while the amount on the charged filter was only 14.3 mg. However, this result differed from the directly measured weight. The weighted dust amount on the charged filter was merely 5.7 mg, approximately 39.8% of the calculated value. We believed this ratio would be lower if the voltage were raised to 40 kV. This phenomenon suggests that the removal of particles by the charged filters may also result from repulsion in addition to interception and electrostatic adsorption. After prolonged filtration testing, the upstream filtration pipe was covered with a substantial amount of dust, peaking in density approximately 10 to 30 cm ahead of the filter, while the downstream filtration pipe remained considerably cleaner.

### 3.6. Performance of Bacterial Removal

**Figure 8:**
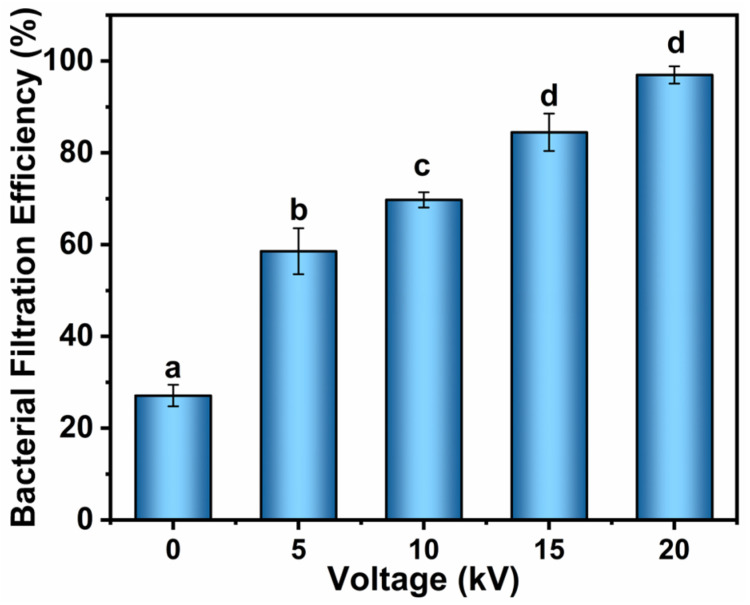
The bacterial filtration efficiency of the WFH-PDA/Ag filter when loaded with HVs.

The bacterial removal performance of the WFH-PDA/Ag filter was evaluated against *E. coli* in Figure 8. The WFH-PDA/Ag filter demonstrated higher bacterial filtration efficiencies with increasing applied voltage. In contrast, the mechanical bacterial filtration efficiency of this filter was only 27.1%. Notably, the BFE values of the WFH-PDA/Ag filters reached 58.6% efficiency at the external HV of 5 kV. As the voltage increased from 5 to 20 kV, the BFE increased linearly by about 10% per 5 kV. At 20 kV, the WFH-PDA/Ag filter achieved a high bacterial filtration efficiency of 97.0%.

Bacterial elimination from the ambient air could be attributed to two possible mechanisms: mechanical blocking and bio-inactivation, with the latter likely being the predominant factor. This hypothesis is supported by two phenomena. First, HV led to a reduction in the number of collected bacteria (Figure S6), indicating that the electrostatic field generated on the filter had an inactivation effect on the bacteria even before they passed through the filter. Second, a considerable number of bacteria were still able to penetrate the filter and deposit on the plates, suggesting that the mechanical interception of the filters had limited efficacy in preventing bacterial deposition.

**Figure 9:**
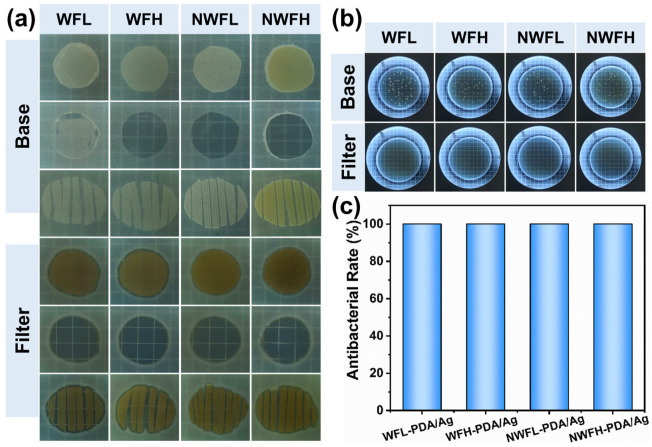
Antibacterial performance of filters with different structures. (**a**) Morphology of antibacterial ozone; (**b**) Bacterial colonies formed on agar plates after contact with filters of different structures; (**c**) Antibacterial rate.

The antibacterial performance of the Base-PDA/Ag filters and substrate filters was investigated in this study. As shown in Figure 9a, the Base-PDA/Ag filters effectively inhibited *E. coli* growth both around and under the filters, whereas bacterial colonies remained visible under the substrate filters. This indicates that the silver coating on the Base-PDA/Ag filters was effective in eliminating bacteria upon contact [49]. Additionally, filters with non-woven fabric (NWF) structures exhibited a relatively high density, which impeded bacterial contact with air and thereby reduced bacterial growth on the agar plates. The results demonstrate that all filters achieved a 100% antibacterial rate, underscoring their effectiveness in preventing bacterial growth.

## 4. Conclusions

In conclusion, this work develops a VLWF system with ultraloose gridding woven filter enhanced by electrostatic charges to achieve effective removal of PM_2.5_ and bacteria while maintaining ultralow air resistance. The key innovations of this VLWF system included the introduction of a robust double-layer woven filter design and the incorporation of the direct high-voltage loading capability into the filters. These features resulted in a remarkably low pressure drop (6.2 Pa) and energy consumption (134 W/m^2^). Once supplied with HVs, the filters functioned as the carriers of an electrostatic field, effectively removing PM_2.5_ through electrostatic repulsion and attraction forces, and while inactivating bacteria by electric field. Furthermore, the double-layer design and pore size reaching up to 73.7 μm rendered the gridding woven filters resistant to clogging and easy to clean, thereby minimizing the need for frequent filter replacements. Given these advantages, we believe that the VLWF system holds great promise for various potential applications, including core components in central ventilation systems, air conditioners, car air purifiers, air sterilizers, refrigerators, or the assembly units for industrial production processes. We anticipate this work will be of interest to a broad spectrum of readers.

## 5. Patents

Patent name: The invention relates to a method for preparing a multi-coated yarn or fabric material with an electrostatic load. Patent number: ZL 201910106584.3.

## Data Availability

Data will be made available on request.

## Supplementary Materials

The following supporting information can be downloaded at https://www.mdpi.com/article/10.3390/polym17131765/s1, Figure S1: Surface specific resistance test; Figure S2: The setup for PM_2.5_ and bacteria filtration tests; Figure S3: Effects of the DOPA modification duration on the appearance of PET-PDA and PET-PDA/Ag fabrics; Figure S4: Appearances of PET-PDA and PET-PDA/Ag filters with different structures; Figure S5: The fiber diameters, thickness, and pore size of filters.

## Abbreviations

The following abbreviations are used in this manuscript:

PET	Polyethylene terephthalate
VLWF	High-voltage loaded woven filtration
WFL	Low density woven fabric
WFH	High density woven fabric
NWFL	Low density nonwoven fabric
NWFH	High density nonwoven fabric
DOPA	Dopamine hydrochloride
HVs	High voltages
PD	Pressure drop
QF	Quality factor
CQF	Comprehensive quality factor
TPA	Total power consumption per unit area
RPs	Removed particles

## References

[1] Tian E., Yu Q., Gao Y., Wang H., Wang C., Zhang Y., Li B., Zhu M., Mo J., Xu G. (2021). Ultralow Resistance Two-Stage Electrostatically Assisted Air Filtration by Polydopamine Coated PET Coarse Filter. Small.

[2] Kim M.W., An S., Seok H., Yarin A.L., Yoon S.S. (2020). Transparent Metallized Microfibers as Recyclable Electrostatic Air Filters with Ionization. ACS Appl. Mater. Interfaces.

[3] Xiang J., Seto E., Mo J., Zhang J., Zhang Y. (2021). Impacts of implementing Healthy Building guidelines for daily PM_2.5_ limit on premature deaths and economic losses in urban China: A population-based modeling study. Environ. Int..

[4] Wahid A., Zhang H., Qin X. (2025). Electrospun nanofiber membranes with micro-gradient structure for highly efficient air filtration applications. Mater. Lett..

[5] Liu F., Wang H., Liu M., Lu Z., Lin Z., Chen Y. (2024). Research on the process of preparing fiber filtration membrane by centrifugal blowing electrospinning technology. Appl. Polym. Sci..

[6] Martins N.R., Graca G.C.D. (2018). Impact of PM_2.5_ in indoor urban environments: A review. Sustain. Cities Soc..

[7] Soares M., Oliveira H., Alves C. (2025). Airborne particulate matter inhalation bioaccessibility: A review of methodological aspects. Chem. Biol. Interact..

[8] Xue T., Kang N., Zhu T. (2025). Health-Oriented Strategy for Clean Air and Climate Actions: Differential Health Effects of Atmospheric Components. Annu. Rev. Public Health.

[9] Dutta S., Bansal P. (2025). Sustainability aspect of wearable clean room filters: A review. Polym. Bull..

[10] Liu H., Cao C., Huang J., Chen Z., Chen G., Lai Y. (2020). Progress on particulate matter filtration technology:Basic concepts, advanced materials, and performances. Nanoscale.

[11] Wang F., Meng D., Li X., Tan J. (2016). Indoor-outdoor relationships of PM_2.5_ in four residential dwellings in winter in the Yangtze River Delta, China. Environ. Pollut..

[12] Rosner D.E., Arias-Zugasti M. (2021). Predicting the aerosol capture characteristics of fibrous filters. I. Exact- and tractable (3-moment) approximate-methods to incorporate aerosol polydispersity effects with a multi-mechanism, semi-analytic single-fiber particle capture fraction. Sep. Purif. Technol..

[13] Abdul-Wahab S., Fadlallah S., Al-Rashdi M. (2018). Evaluation of the impact of ground-level concentrations of SO_2_, NO_x_, CO, and PM_10_ emitted from a steel melting plant on Muscat, Oman. Sustain. Cities Soc..

[14] Tian E., Mo J. (2019). Toward energy saving and high efficiency through an optimized use of a PET coarse filter: The development of a new electrostatically assisted air filter. Energy Build.

[15] Zhao K., Huang J., Mao J., Bao Z., Chen Z., Lai Y. (2020). Charged graphene aerogel filter enabled superior particulate matter removal efficiency in harsh environment. Chem. Eng..

[16] Wang Q., Khan F., Wei L., Shen H., Zhang C., Jiang Q., Qiu Y. (2017). Filtration properties of carbon woven fabric filters supplied with high voltage for removal of PM 1.0 particles. Sep. Purif. Technol..

[17] Zhang X., Wang Y., Liu W., Jin X. (2022). Needle-punched electret air filters (NEAFs) with high filtration efficiency, low filtration resistance, and superior dust holding capacity. Sep. Purif. Technol..

[18] Li C.L., Song W.Z., Sun D.J., Zhang M., Zhang J., Chen Y.Q., Ramakrishna S., Long Y.Z. (2023). A self-priming air filtration system based on triboelectric nanogenerator for active air purification. Chem. Eng..

[19] Kim M.W., An S., Seok H., Yoon S.S., Yarin A.L. (2019). Electrostatic Transparent Air Filter Membranes Composed of Metallized Microfibers for Particulate Removal. ACS Appl. Mater. Interfaces.

[20] Joubert A., Abd Ali S.A.Z., Frossard M., Andrès Y. (2021). Dust and microbial filtration performance of regular and antimicrobial HVAC filters in realistic conditions. Environ. Sci. Pollut. Res..

[21] Jafari M., Shim E., Joijode A. (2021). Fabrication of Poly(lactic acid) filter media via the meltblowing process and their filtration performances: A comparative study with polypropylene meltblown. Sep. Purif. Technol..

[22] Guo J., Hanif A., Shang J., Deka B.J., Zhi N., An A.K. (2021). PAA@ZIF-8 incorporated nanofibrous membrane for high-efficiency PM2.5 capture. Chem. Eng..

[23] Wang G., Sun L., Zhao B., Fang Y., Qi Y., Ning G., Ye J. (2023). Reusable Electrospun Nanofibrous Membranes with Antibacterial Activity for Air Filtration. ACS Appl. Nano Mater..

[24] Kim S., Sioutas C., Chang M. (2000). Electrostatic Enhancement of the Collection Efficiency of Stainless Steel Fiber Filters. Aerosol Sci. Technol..

[25] Choi D.Y., Jung S.-H., Song D.K., An E.J., Park D., Kim T.-O., Jung J.H., Lee H.M. (2017). Al-Coated Conductive Fibrous Filter with Low Pressure Drop for Efficient Electrostatic Capture of Ultrafine Particulate Pollutants. ACS Appl. Mater. Interfaces.

[26] Zhao P., Riesch J., Höschen T., Almanstötter J., Balden M., Coenen J.W., Himml R., Pantleon W., Toussaint U.V., Neu R. (2017). Microstructure, mechanical behaviour and fracture of pure tungsten wire after different heat treatments. Int. J. Refract. Met. Hard Mater..

[27] Wang C., Jiang J., Wang P., Kong L., Liu J. (2025). Exploring the potential of a novel electrostatic precipitator as an alternative to air filters in air purifiers. Build Environ..

[28] Güler O., Varol T., Alver Ü., Çanakçı A. (2019). The effect of flake-like morphology on the coating properties of silver coated copper particles fabricated by electroless plating. J. Alloys Compd..

[29] Kim Y.I., Kim M.W., An S., Yarin A.L., Yoon S.S. (2020). Reusable Filters Augmented with Heating Microfibers for Antibacterial and Antiviral Sterilization. ACS Appl. Mater. Interfaces.

[30] Liu G., Zhou N., Xiong Q. (2021). Preparation of highly conductive and flexible Ag-coated single fiberglass via dopamine functionalization and electroless depositing. Mater. Sci. Mater. Electron.

[31] Cui A., Yao J., Xu J., Wang R., Hao L. (2024). Fabricating durable conductive coatings from PEDOT: PSS/Ag composite and PDA by sustainable ink-jet printing on cotton fabric. Prog. Org. Coat..

[32] Sun J., Hu J., Liang M., Gao L., Cao H., Liu T., Zhao Y., Wei J., Zhang H., Wang H. (2025). Rapid preparation of mussel-inspired coatings with adjustable properties under electrochemical drive. Surf. Interfaces.

[33] Tan S., Song S., Pang L., Zhang X., Xu R., Zheng J., Zhao S., Li L. (2025). Construction of effective conductive network of the core-shell SiO_2_ microspheres hybrid MWCNTs in NR matrix. Compos. Commun..

[34] Ren X., Hao R., Yang Y., Zhao G., Liu Y., Duan H. (2023). A facile and green strategy to achieve metallized woven carbon fiber through the triple roles of dopamine in in-situ thermal reduction of Ag. Compos. Commun..

[35] Duygulu N.E., Altinbay A., Ciftci F. (2024). Antibacterial, Mechanical, and Thermal Properties of Ag, ZnO, TiO_2_ Reinforced PVA Nanocomposite Fibers. ChemistrySelect.

[36] Wang H., Zhou H., Liu S., Shao H., Fu S., Rutledge G.C., Lin T. (2017). Durable, self-healing, superhydrophobic fabrics from fluorine-free, waterborne, polydopamine/alkyl silane coatings. RSC Adv..

[37] Chen Y., Wu X., Wei J., Wu H. (2020). Characterization and Application to Fiber Reinforced Composite of Catechol/polyethyleneimine Modified Polyester Fabrics by Mussel-inspiration. Fibers Polym..

[38] Li Q., Zhang S., Mahmood K., Jin Y., Huang C., Huang Z., Zhang S., Ming W. (2021). Fabrication of multifunctional PET fabrics with flame retardant, antibacterial and superhydrophobic properties. Prog. Org. Coat..

[39] Du J., Jing C. (2019). One-step fabrication of dopamine-inspired Au for SERS sensing of Cd^2+^ and polycyclic aromatic hydrocarbons. Anal. Chim. Acta.

[40] Zhu S., Gu Z., Xiong S., An Y., Liu Y., Yin T., You J., Hu Y. (2016). Fabrication of a novel bio-inspired collagen–polydopamine hydrogel and insights into the formation mechanism for biomedical applications. RSC Adv..

[41] Wang W., Cheng W., Tian M., Zou H., Li L., Zhang L. (2012). Preparation of PET/Ag hybrid fibers via a biomimetic surface functionalization method. Electrochim. Acta.

[42] Wang N., Yang Y., Al-Deyab S.S., El-Newehy M., Yu J., Ding B. (2015). Ultra-light 3D nanofibre-nets binary structured nylon 6–polyacrylonitrile membranes for efficient filtration of fine particulate matter. Mater. Chem..

[43] Zhang X., Ma J., Nie X., Fan Y., Wang H., Cui Y. (2023). Establishment of air fiber filtration model based on fractal theory and analysis of filtration performances. Mater. Today Commun..

[44] Lee K.S., Hasolli N., Lee J.R., Kim K.D., Kim S.D., Park Y.O., Hwang J. (2020). Dust loading performance of a non-electret HVAC filter module in the presence of an external electric field. Sep. Purif. Technol..

[45] Tian E., Mo J., Li X. (2018). Electrostatically assisted metal foam coarse filter with small pressure drop for efficient removal of fine particles: Effect of filter medium. Build Environ..

[46] Standard Test Method for Characterizing the Pressure Drop and Filtration Performance of Cleanable Filter Media. https://store.astm.org/d6830-02.html.

[47] Feng Z., Long Z., Mo J. (2016). Experimental and theoretical study of a novel electrostatic enhanced air filter (EEAF) for fine particles. Aerosol Sci..

[48] Tian E., Xia F., Wu J., Zhang Y., Li J., Wang H., Mo J. (2020). Electrostatic Air Filtration by Multifunctional Dielectric Heterocaking Filters with Ultralow Pressure Drop. ACS Appl. Mater. Interfaces.

[49] Zhang S., Jiang W., Liu G., Liu S., Chen H., Lyu G., Yang G., Liu Y., Ni Y. (2023). Preparation of ultrafine and highly loaded silver nanoparticle composites and their highly efficient applications as reductive catalysts and antibacterial agents. Colloid Interface Sci..

