# Supplementary material for: Ultralow-Resistance High-Voltage Loaded Woven Air Filter for Fine Particle/Bacteria Removal
Source: Polymers (Basel). 2025 Jun 26;17(13):1765. doi: 10.3390/polym17131765 (PMC12251714; doi:10.3390/polym17131765)
Supplement: Supplementary file 1 [file polymers-17-01765-s001.zip › polymers-3613720-supplementary.pdf]

Supplementary Information

Ultralow-resistance high-voltage loaded woven air filter for fine particle/bacteria removal

Weisi Fan [1,2], Sanqiang Wei [3], Ziyun Zhang [1,2], Lulu Shi [1,2], Jun Wang [1,2], Wenlan Hao [3], Kun Zhang [1,2] and Qiuran Jiang [1,2,*]

[1] *Key Laboratory of Textile Science &Technology, Ministry of Education, College of Textiles, Donghua University, Shanghai 201620, China;*

[2] *Department of Technical Textiles, College of Textiles, Donghua University, Shanghai 201620, China*

[3] *Beijing Jinmao Living Environment Technology Co., Ltd., Beijing 102629, China;*

* Corresponding Author: Qiuran Jiang, RM 4044, NO.3 College Building, 2999 North Renmin Road, Songjiang District, Shanghai 201620, P. R. China. Tel: +86-21-67792380; Fax: +86-21-67792627; Email: jj@dhu.edu.cn

Materials and Methods

1.1 Materials

The base substrates were polyethylene terephthalate (PET) fabrics with four structures including two nonwoven fabrics with different fabric weights designated as samples NWFL (55 g/m^2) and NWFH (113 g/m^2), two monofilament plain gridding woven fabrics with distinct fabric densities assigned as samples WFL (35 g/m^2, thread count 489 × 484 ends/10 cm) and WFH (43 g/m^2, thread count 822 × 800 ends/10 cm). These fabrics were provided by Jiaxing Yitaile Electronics Co., Ltd. Dopamine hydrochloride (DOPA) was purchased from Beijing Bailingwei Technology Co., Ltd. Silver nitrate was acquired from Shanghai Lingfeng Chemical Reagent Co., Ltd. The gram-negative bacteria, Escherichia coli (E. coli, ATCC 25922 strain) were bought from Shanghai Preservation Biotechnology Center. Other chemicals were bought from Sinopharm Chemical Reagent Co., Ltd or Shanghai Biological Engineering Co., Ltd. All reagents were of analytical grade and used as received.

1.2 Surface conductivity test

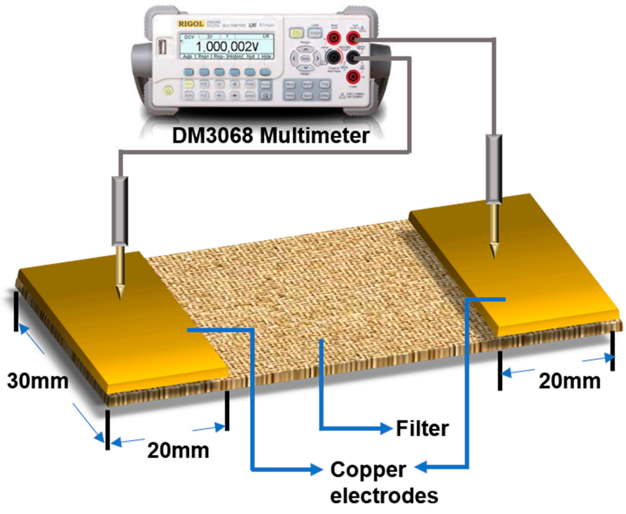

Figure S1 Surface specific resistance test.

The process for testing the surface specific resistance of filters is illustrated in **Figure S1**. The test sample was prepared with dimensions of 70 mm × 35 mm to ensure the complete contact with the copper electrodes (20 mm × 30 mm). The two electrodes were located at the two edges of filters as shown in Figure S1. The probes of a multimeter (DM3068, RIGOL, Beijing, China) were connected to the electrodes. The multimeter was set to the resistance measurement mode and the resistance values were recorded after balancing for 2 min. Each sample was tested for 10 times, and the surface specific resistance was calculated using the following equation:

$$\rho_s = \frac{W}{L} \times R_s \quad \text{(S1)}$$

where ρ_s is the surface specific resistance (Ω/m^2), w is the width of the sample (mm), L is the length of the sample (mm), R_s is the resistance measured by a probe (Ω/m^2).

1.3 Ozone test

Given the application of high voltages (HVs) on filters, the potential generation of harmful ozone is a concern. The ozone generation of the entire filtration system, comprising a negatively charged ionizer (Yufeng Electronics Co., Ltd., China), a HV-loaded filter, and a grounding filter, was measured. The woven filter (WFH-PDA/Ag) and the negatively charged ionizer (-3.5 kV) were placed in a sealed chamber (350 L) and subjected to a HV of 20 kV for 8 h. The accumulated ozone concentrations were measured using an ozonemeter (SKS-BA-03, Guangdong Si Kesen gas detection Ltd.)

1.4 Antibacterial performance test

The antibacterial performance of the filters was investigated using the agar diffusion plate method and the shake flask method following the standards GB/T 20944.1 and 20944.3. The Escherichia coli (E. coli, ATCC 25922) was selected as the model microorganism. A single

colony of E. coli was inoculated into the 20 mL Luria Bertani (LB) broth (1 g/100 mL tryptone, 0.5 g/100 mL yeast extract, and 0.5 g/100 mL NaCl) for 18 h with shaking at 130 rpm to obtain the bacterial suspension. The optical density of E. coli suspension was measured by a UV-Vis spectrophotometer (Lambda35, Perkin Elmer, USA) at the wavelength of 600 nm (OD_{600}). The stationary phase was reached when the OD_{600} value of the bacterial suspension increased to 0.8. The E. coli suspension was centrifuged at 2500 rpm for 10 min. The broth was replaced with sterilized PBS solution, and the E. coli was resuspended. This washing process was repeated three times. The desired bacterial concentration is illustrated in the following sections.

The quantitative antibacterial test employing the shake flask method involved the following detailed procedures: 0.1 g of substrate fabrics and fabric index-PDA/Ag filters were placed into conical flasks containing 9 mL PBS solution. The bacteria suspension was diluted to approximately 10^5 colony-forming units per milliliter (CFU/mL) using PBS solution. A 1 mL aliquot of the bacterial suspension was transferred to each conical flask and shaken at a speed of 150 rpm at 24°C for 18 h. After shaking, the solution from each flask (1 mL) was pipetted out and subjected to a series of gradient dilutions. The pure LB agar plates were smeared with 200 μL solution after a series of gradient dilutions and incubated at 37°C for 24 h. The colonies formed on the agar plates were counted, and the antibacterial rate was calculated by the following equation:

$$Antibacterial\ rate\ (\%) = \left(1 - \frac{CFU_{experiment}}{CFU_{control}}\right) \times 100\% \quad (S2)$$

where $CFU_{experiment}$ and $CFU_{control}$ are the colonies formed on the agar plate that were taken out from the bacterial suspension after contacting with the substrate fabrics and fabric index-PDA/Ag filters, respectively.

In the agar diffusion plate test, 10 mL of sterilized LB agar was first poured into petri dishes. A bacterial suspension (1 mL, 10^8 CFU/mL) was then inoculated into 150 mL of sterilized LB agar at 45°C and poured into prepared petri dishes (5 mL/dish). Circular substrate fabric and fabric index-PDA/Ag filters, each with a diameter of 2.5 cm, were placed on the surface of the agar plate until the agar cooled to room temperature. The plates were incubated at 37°C for 24 h, after which images of the inhibitory zones around and beneath the specimens were captured. All antibacterial tests were conducted in triplicate.

1.5 $PM_{2.5}$ filtration performance test

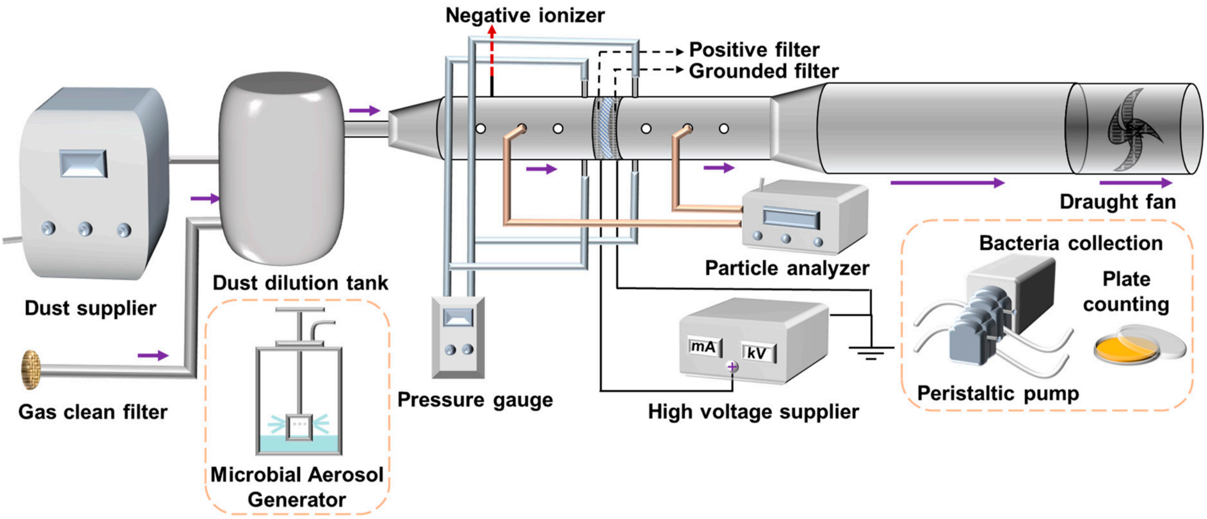

Figure S2 The setup for $PM_{2.5}$ and bacteria filtration tests.

The $PM_{2.5}$ filtration performance of filters was tested using a customized filtration testing system. This system was equipped with a dust supplying subsystem (dust supplier (RGB1000, PALAS®, Germany), dust dilution tank and clean gas supplying pipes), a testing channel with an internal diameter: 12 cm, a multi-function ventilation meter (9565-P VeloclCalc, TSI), and two portable real-time aerosol spectrometers (Fidas®Frog, PALAS®, Germany) (**Figure S2**). In the filtration testing channel, the filters (diameter: 13 cm) were mounted on the active sample

frame, which was connected to a high-voltage power supply (ES50P-10W/DDPM, GAMMA, USA), and a grounding frame was positioned 5 cm behind the sample frame. A negative ionizer (Yufeng Electronics co., Ltd., China) was mounted in front of the sample frame. Pre-dried standard dusts (PTI's ISO 12103-1, A1 Ultrafine Test Dust) were loaded into the dust supplier and dispersed into the dust dilution tank at a feeding rate of 1 mm/h. The mixture was balanced to a stable concentration with clean air. The dust dispersion was introduced into the testing channel at an initial concentration of 500 μg/m^3 with a face velocity of 0.1 m/s. During the tests, the ionizer was supplied with negative high voltage at 3.5 kV, and the sample frame was provided with positive high voltages ranging from 0 to 40 kV. The $PM_{2.5}$ concentrations before and after filtration were measured using a portable real-time aerosol spectrometer (Fidas®Frog, PALAS®, Germany). Filtration efficiency was calculated according to the following equation:

$$\mathrm{E} = \left(1 - \frac{C_{after}}{C_{before}}\right) \times 100\% \tag{S3}$$

where E is the filtration efficiency, C_{before} and C_{after} are the particulate concentrations before and after filtration (particles/cm^3). The air pressure drop (ΔP) was obtained by a multi-function ventilation meter. The quality factor of each filter was also calculated based on the following equation:

$$\mathrm{QF} = -\frac{\ln(1-\mathrm{E})}{\Delta p} \tag{S4}$$

where QF is the quality factor (Pa^{-1}), E is the filtration efficiency, ΔP is the pressure drop (Pa), and is abbreviated as PD in subsequent.

1.6 Dust accumulation evaluation

The behavior of dust accumulation on filters was investigated to elucidate the filtration mechanism. To facilitate observation, filters with relatively compact structures (NWFH and

WFH-PDA/Ag filters) were utilized for testing and fixed to the sample frames in the customized filtration testing channel. Dust was introduced into the channel at a uniform concentration and velocity for the filtration test. The filters were supplied with a high voltage of 20 kV. After operating for 5 h, the filters were sputter-coated with gold and observed under a scanning electron microscope (SEM, TM3000, Hitachi, Japan). To quantitatively assess the dust accumulation behavior of filters, standard dust was supplied into the testing channel at a higher average concentration of 1.75 mg/m^3. The face velocity was maintained at 0.1 m/s. The weight changes of the filters after 3 h of operation were measured and compared with the theoretical accumulated dust weights, calculated by the following equation:

$$M_c = T_f \cdot E \cdot C_d \cdot V \cdot \pi \times \left(\frac{D}{2} \times 10^{-2}\right)^2 \tag{S5}$$

where M_c is the theoretical mass of accumulated dust (mg), T_f is the filtration duration (s), E is the filtration efficiency (%), C_d is the dust concentration (mg/m^3), V is the face velocity (m/s), and D is the diameter of the test pipe (cm).

1.7 Bacteria filtration performance test

The bacterial suspension was prepared as described in section 1.4, with a final concentration of approximately 10^6 CFU/mL. The filters were mounted on the filter frame and supplied with high voltages. The air flow rate was maintained at 0.1 m/s. The bacteria suspension (20 mL) was loaded into a microbial aerosol generator (TK-3, Beijing Zhongxi Yuanda Technology Co., Ltd., China) and aerosolized into the filtration channel at a rate of 0.3 mL/min. Bioaerosols were collected 30 cm downstream of the filter using an Andersen six-stage impactor sampler (FA-1, Weifang Aiwo Environmental Protection Equipment Co., Ltd, China). LB agar plates were loaded in the impactors and pumped the air from the filtration channel at a flow rate of 14

L/min for 5 min. The collected agar plates were incubated at 37°C for 24 h. The number of colonies in each plate was counted. The bacteria filtration efficiency (BFE) was calculated by the following equation:

$$\mathrm{BFE} = \left(1 - \frac{CFU_{after}}{CFU_{before}}\right) \times 100 \tag{S6}$$

where $\mathrm{CFU}_{\mathrm{before}}$ and $\mathrm{CFU}_{\mathrm{after}}$ are the summation of all colony forming units on the agar plates over impactor stages 1–6 cultured with the E. coli suspensions collected without a filter and with a filter under different voltages.

1.8 Electric field simulation

Three-dimensional models of woven filters were established using Solidworks (version 2018) based on parameters such as fiber diameters, fabric densities, and thicknesses. COMSOL Multiphysics 5.5 was utilized to simulate the electric fields around the filters. The electrostatics module within the AC/DC module was selected, with a stationary study chosen under general studies. Regions with a radius of 7.5 cm and an acrylic circular pipe with a length of 120 cm were created to match the experimental settings. Due to the computational complexity as the number of fibers in the filter increases, the filter model is simplified. For the macroscopic electric field simulation on the filter's side, the filter was modeled as a cylinder with a radius of 7.5 cm and a thickness of 0.09 mm, and the material was set to silver. The negative ion, characterized by its dimensions, was modeled as a cone with a base radius of 0.4 mm, a top radius of 0.9 mm, and a height of 8 mm, and the material was indicative of tungsten. The negative ionizer generator was positioned 45 cm ahead of the positive filter. The voltage supplied to the active filter ranged from 0 to 40 kV, whereas the grounded filter remained grounded. To simulate the electric fields on the surface of the active filters, circular fabric

regions with a radius of 0.6 mm and varying yarn densities were constructed.

1.9 Statistical analysis

All the data were analyzed using one-way analysis of variance (ANOVA) with Turkey's pairwise multiple comparison test. A 95% confidence interval was established. Results were considered statistically significantly different if the *p*-value was less than 0.05, and different letters were used to denote significant differences in graphs.

2 Filter appearance

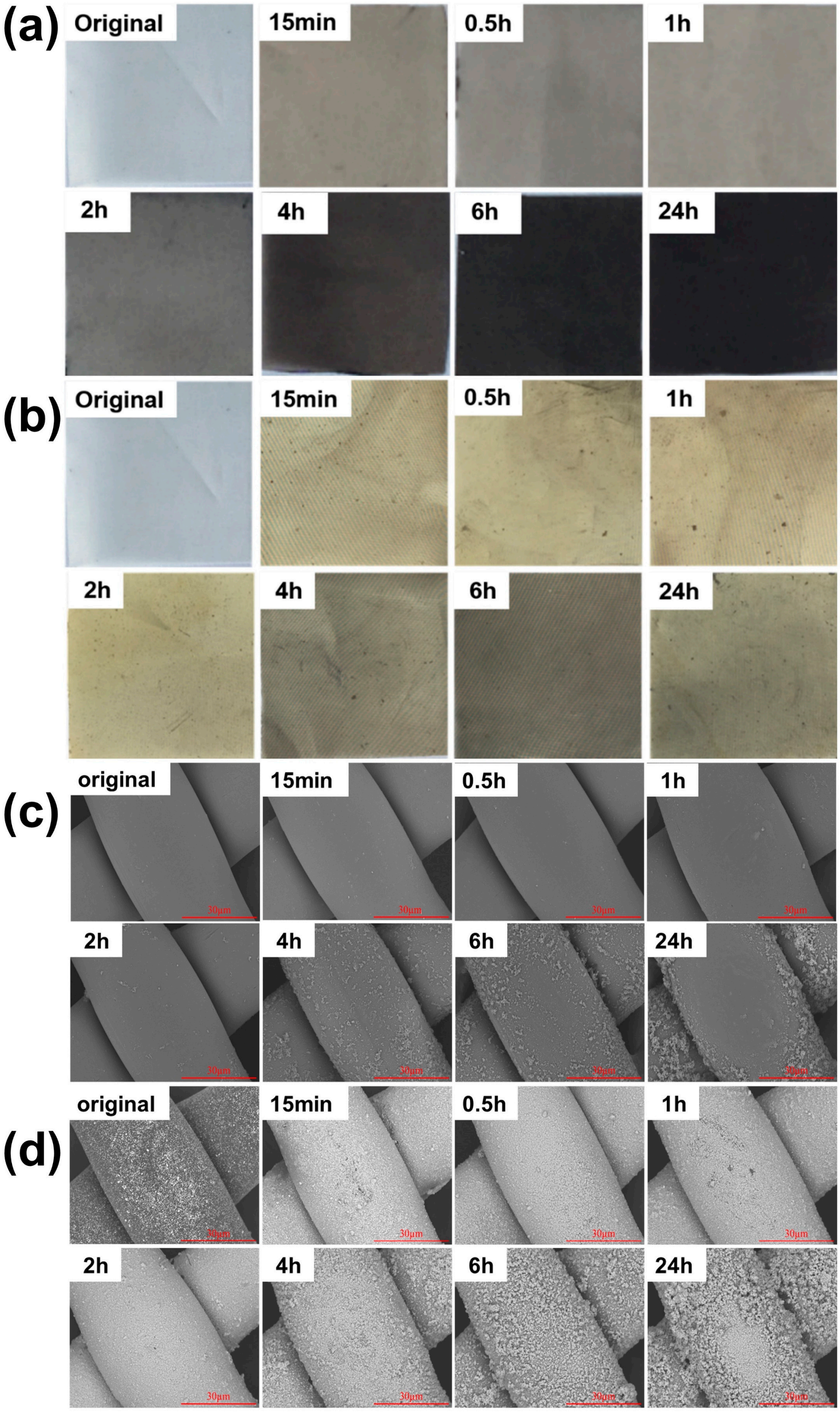

Figure S3 Effects of the DOPA modification duration on the appearance of PET-PDA (a, c) and PET-PDA/Ag fabrics (b, d).

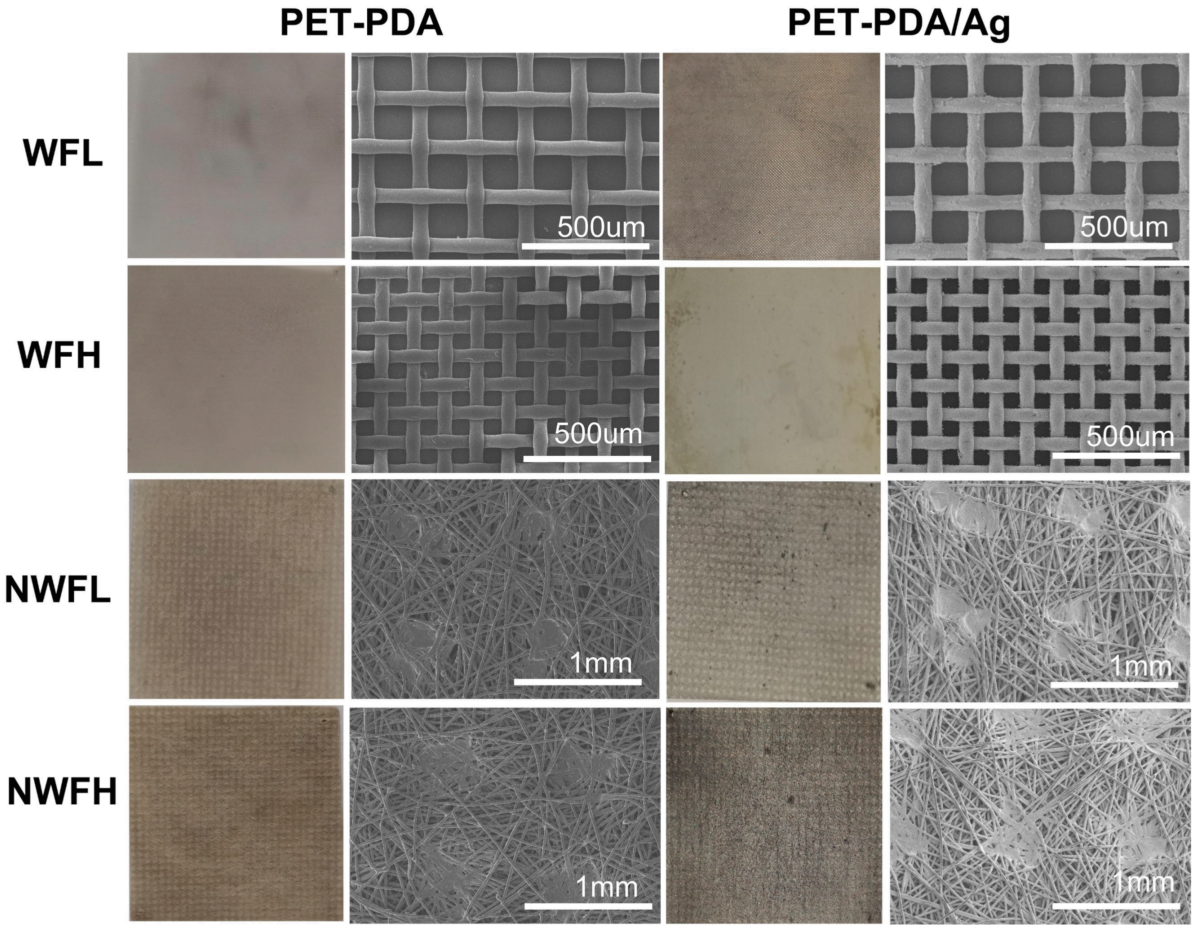

Figure S4 Appearances of PET-PDA and PET-PDA/Ag filters with different structures.

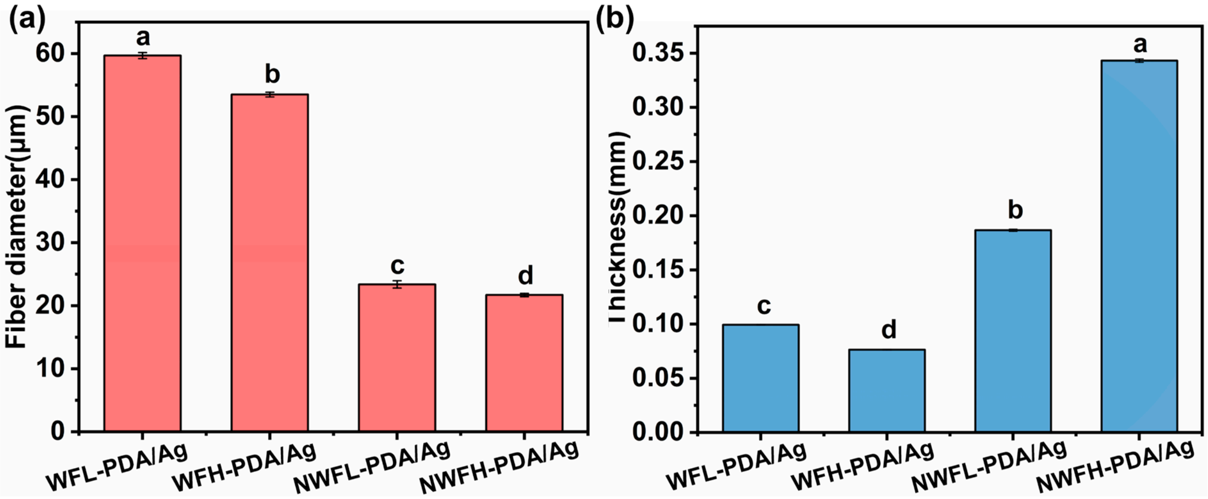

(c)

Fabric Type	Pore size (μm)
WFL-PDA/Ag	148.3
WFH-PDA/Ag	73.7
NWFL-PDA/Ag	33.5
NWFH-PDA/Ag	26.0

Figure S5 The fiber diameters (a), thickness (b), and pore size of filters (c).

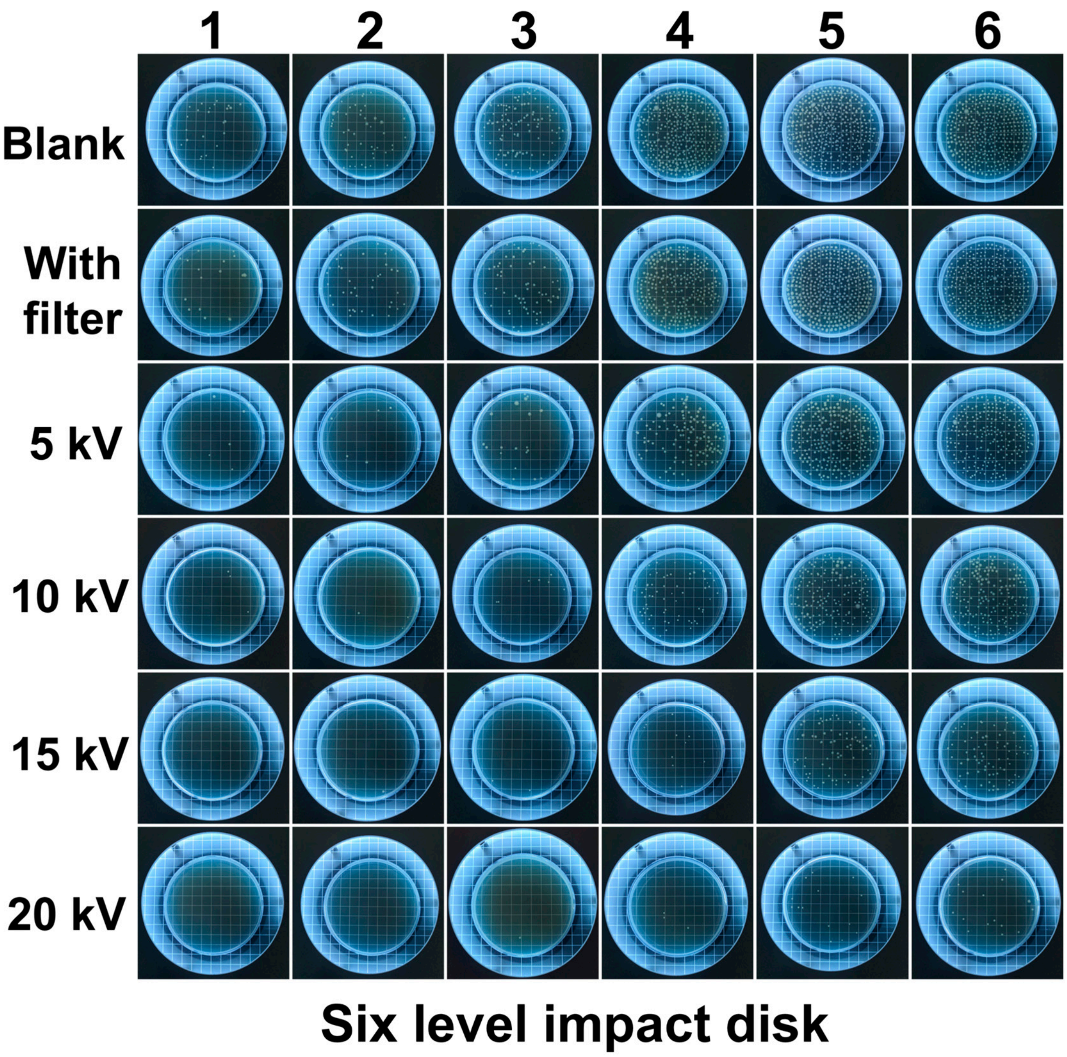

Figure S6 The colony units formed on the culture dishes when WFH-PDA/Ag filter loaded with different voltages.

3 Parameters for filtration performance evaluation

The calculation of total power consumption per unit area (TPA) and the comprehensive quality factor (CQF) was conducted according to the procedures outlined in the literature[1, 2]. The device power dissipation, denoted as P_d (W/m^2), was computed using the following equation:

$$P_d = \frac{U_c \cdot I_c + U_p \cdot I_p}{A} \tag{S7}$$

where U_c and U_p (kV) represent the voltages applied to the negative pre-charger and the positive

filter, respectively, while I (mA) denotes the current in the respective section, and A (m^2) signifies the cross-sectional area. The calculation of total power consumption per unit area, denoted as Pt (W/m^2), was calculated using the following equation:

$$P_t = P_d + P_f = P_d + \frac{W_f}{A} = P_d + \frac{\Delta p \cdot Q}{A \cdot \eta_{fan}} = P_d + \frac{\Delta p \cdot V_{air}}{\eta_{fan}} \tag{S8}$$

where P_f (W/m^2) corresponds to the power dissipation of the fan, W_f (W) represents the power required to overcome filter flow resistance, Q (m^3/s) is the volumetric air flow rate, V_{air} (m/s) signifies the face air velocity through the filters, and η_{fan} =0.71 is the overall fan efficiency. The comprehensive quality factor (CQF， Pa^{-1}) was determined via the following equation:

$$CQF = \frac{-\ln(1 - E)}{\Delta p + \frac{\eta_{fan} \cdot P_d}{V_{air}}} \tag{S9}$$

where E is the filtration efficiency, ΔP is the pressure drop, η_{fan} =0.71 is the overall fan efficiency, P_d is the device power dissipation and V_{air} is the face air velocity through the filters.

4 Statistical analysis results

Table S1 Filtration performance statistical analysis

Voltage	Sample	Filtration Efficiency (%)	Quality Factor (Pa^{-1})	Comprehensive Quality Factor (Pa^{-1})
0 kV	WFL	a	a	a
	WFH	a	b c d	a b
	NWFL	b d	c	a b c
	NWFH	c h j	c d	c d e
10 kV	WFL	d	e	b c d e
	WFH	b d	a b	b c e

Table S1 Filtration performance statistical analysis (continued)

	NWFL	c e h i	a b	f h j k
	NWFH	f k	a b c d	f g j k
20 kV	WFL	b g	f	d e h i
	WFH	b g	a	d e i
	NWFL	h j	a b c d	d h i
	NWFH	c e i	b c d	d e h i
30 kV	WFL	g	f g	h i
	WFH	j	h	h j k
	NWFL	e k	a	f g
	NWFH	f	a b d	f g
40 kV	WFL	g	g	h i k
	WFH	c h i	e	f g
	NWFL	e i k	a	f g j
	NWFH	f	a b	g

References

[1] Tian E, Mo J. Toward energy saving and high efficiency through an optimized use of a PET coarse filter: The development of a new electrostatically assisted air filter [J]. Energy and Buildings, 2019, 186: 276-283. http://doi.org/10.1016/j.enbuild.2019.01.021.

[2] Tian E, Mo J, Li X. Electrostatically assisted metal foam coarse filter with small pressure drop for efficient removal of fine particles: Effect of filter medium [J]. Building and Environment, 2018, 144: 419-426. http://doi.org/10.1016/j.buildenv.2018.08.026.
